# Inhibition of DNA and Histone Methylation by 5-Aza-2′-Deoxycytidine (Decitabine) and 3-Deazaneplanocin-A on Antineoplastic Action and Gene Expression in Myeloid Leukemic Cells

**DOI:** 10.3389/fonc.2017.00019

**Published:** 2017-02-15

**Authors:** Richard L. Momparler, Sylvie Côté, Louise F. Momparler, Youssef Idaghdour

**Affiliations:** ^1^Département de Pharmacologie, Université de Montréal, Montreal, QC, Canada; ^2^Centre de recherche, Service d’hématologie/oncologie, CHU-Saint-Justine, Montréal, QC, Canada; ^3^Department of Biology, New York University Abu Dhabi, Abu Dhabi, United Arab Emirates

**Keywords:** 5-aza-2-deoxycytidine, 3-deazaneplanocin A, myeloid leukemia, epigenetics, gene expression, chemotherapy

## Abstract

Epigenetic alterations play an important role in the development of acute myeloid leukemia (AML) by silencing of genes that suppress leukemogenesis and differentiation. One of the key epigenetic changes in AML is gene silencing by DNA methylation. The importance of this alteration is illustrated by the induction of remissions in AML by 5-aza-2′-deoxycytidine (5-AZA-CdR, decitabine), a potent inhibitor of DNA methylation. However, most patients induced into remission by 5-AZA-CdR will relapse, suggesting that a second agent should be sought to increase the efficacy of this epigenetic therapy. An interesting candidate for this purpose is 3-deazaneplanocin A (DZNep). This analog inhibits EZH2, a histone methyltransferase that trimethylates lysine 27 histone H3 (H3K27me3), a marker for gene silencing. This second epigenetic silencing mechanism also plays an important role in leukemogenesis as shown in preclinical studies where DZNep exhibits potent inhibition of colony formation by AML cells. We reported previously that 5-AZA-CdR in combination with DZNep exhibits a synergistic antineoplastic action against human HL-60 AML cells and the synergistic activation of several tumor suppressor genes. In this report, we showed that this combination also induced a synergistic activation of apoptosis in HL-60 cells. The synergistic antineoplastic action of 5-AZA-CdR plus DZNep was also observed on a second human myeloid leukemia cell line, AML-3. In addition, 5-AZA-CdR in combination with the specific inhibitors of EZH2, GSK-126, or GSK-343, also exhibited a synergistic antineoplastic action on both HL-60 and AML-3. The combined action of 5-AZA-CdR and DZNep on global gene expression in HL-60 cells was investigated in greater depth using RNA sequencing analysis. We observed that this combination of epigenetic agents exhibited a synergistic activation of hundreds of genes. The synergistic activation of so many genes that suppress malignancy by 5-AZA-CdR plus DZNep suggests that epigenetic gene silencing by DNA and histone methylation plays a major role in leukemogenesis. Targeting DNA and histone methylation is a promising approach that merits clinical investigation for the treatment of AML.

## Introduction

Acute myeloid leukemia (AML) is the most common type of acute leukemia in adults (1). Despite the use of intensive chemotherapy and stem cell transplantation, AML is still fatal in approximately half of younger patients and in about 80% of elderly patients as a result of primary drug refractoriness, relapse, or treatment-related mortality (2). Consequently, there is an urgent need to develop more specific and effective drugs to target leukemia-specific alterations. Both genetic and epigenetic alterations play an important role in leukemogenesis (3). Epigenetics is defined as heritable changes in gene expression that is not caused by changes in the DNA sequence. These changes involve primarily DNA methylation, histone modifications, chromatin remodeling, and microRNAs.

A frequent epigenetic alteration that is observed in AML is aberrant DNA methylation (4–7). This event occurs by the conversion of cytosine to 5-methylcytosine by DNA methyltransferase in the promoter region of genes. The genes silenced by DNA methylation include tumor suppressors, transcription factors, apoptosis-related proteins, and regulators of myeloid differentiation (7). The importance of this epigenetic alteration is shown by the successful use of inhibitor of DNA methylation (5-aza-2′-deoxycytidine, decitabine, 5-AZA-CdR) to induce complete remissions in patients with AML (8–10). However, therapy of AML with 5-AZA-CdR is not curative (5, 11, 12).

It may be possible to increase the effectiveness of epigenetic therapy by using combinations of epigenetic agents. Chemical modifications of histones can also produce epigenetic changes in gene expression. The initial discovery that a histone deacetylase (HDAC) inhibitor enhanced gene expression of 5-AZA-CdR on leukemic cells (13) provided the rationale to investigate combinations of these two classes of epigenetic agents in clinical trials. This approach was supported by preclinical studies that showed the synergistic interaction of 5-AZA-CdR and the HDAC inhibitor, sodium phenylbutyrate, on leukemic cells and in an animal model of leukemia (14). Interesting clinical responses were observed in AML patients treated with 5-AZA-CdR and the HDAC inhibitor, valproic acid (5). However, these combinational chemotherapies did not improve the long-term outcome of AML (5), indicating that other approaches should be investigated.

An interesting target for epigenetic therapy is the histone methyltransferases EZH2 (15, 16). EZH2 is a subunit of the polycomb repressive complex 2. It plays a key role in embryonic development by suppression of the genes for differentiation so that embryonic stem cells maintain their “self-renewal” capacity and pluripotency (17). EZH2 trimethylates lysine 27 on histone 3 (H3K27me3), a repressive marker for gene expression. Several studies have shown that EZH2 can function as an oncogene. Knockdown of EZH2 protein by RNA interference results in growth inhibition in several tumor models (18). EZH2 overexpression has been reported in several types of cancer and is associated with adverse outcomes (19). The overexpression of EZH2 can be due to gene amplification (20) or a gain-in-function mutation (21).

In this report, we have selected 3-deazaneplanocin-A (DZNep) to target EZH2 and investigated its mechanism and capacity to enhance the antineoplastic action of 5-AZA-CdR. DZNep inhibits S-adenosylhomocysteine hydrolase, resulting in the cellular accumulation of S-adenosylhomocysteine, which acts as a competitive inhibitor of S-adenosyl-l-methionine, the methyl donor for methyltransferases (22). DZNep is a global inhibitor of methyltransferases (23). The preferential target of DZNep is EZH2 as shown by the reduction in the level of this enzyme and H3K27me3 after treatment of tumor cells with this analog (24). DZNep also induces apoptosis of tumor cells. DZNep activates developmental genes that are not silenced by DNA methylation (23, 24). Other EZH2 inhibitors that target the catalytic site of this enzyme are under investigation and show promising clinical activity against lymphoma (15, 16).

The rationale to use DZNep in combination with 5-AZA-CdR for the therapy of AML is as follows: first, DZNep as a single agent shows activity against AML cells as indicated by the reduction in colony formation, induction of differentiation and increase survival of mice with AML (25). Second, some genes silenced by DNA methylation can escape gene activation by 5-AZA-CdR if they contain the repressive marker H3K27me3 (26). Third, genes that program differentiation and marked with H3K27me3 in embryonic stem cells have the highest probability to undergo DNA methylation in cancers (27). Fourth, a “cross-talk” exists between histone and DNA methylation. The trimethylation of H3K27 by EZH2 pre-marks genes for DNA methylation in cancer (28–31). These observations suggest that the interaction between DNA and histone methylation (H3K27me3) can promote leukemogenesis by silencing the genes that program differentiation. Targeting these two gene epigenetic silencing mechanisms using 5-AZA-CdR and DZNep has the potential to reverse this process and lead to effective therapy of AML (32).

We have reported previously that 5-AZA-CdR in combination with DZNep exhibits a synergistic antineoplastic action against AML cells and the reactivation of several genes that suppress leukemogenesis (33). In this report, in order to fully understand the chemotherapeutic potential of DZNep in combination with 5-AZA-CdR for the treatment of AML, we have investigated in depth their activation of a large number of genes that can suppress leukemogenesis using RNA sequence analysis. In addition, we demonstrated that their antineoplastic action was related to the induction of apoptosis and their synergistic antileukemic action was also observed with a second human myeloid leukemic cell line, AML-3 (34).

## Materials and Methods

### Cell Lines and Materials

Human HL-60 myeloid leukemic cells were obtained from ATCC. The HL-60 cells were maintained in RPMI-1640-HEPES containing 10% fetal bovine serum (Wisent). Human OCI-AML-3 (AML-3) myeloid leukemic cells were kindly provided by Mark Minden, Princess Margaret Hospital, Toronto. The AML-3 cells were maintained in alpha-MEM containing 10% fetal bovine serum (Wisent). 5-AZA-CdR was obtained from Dr. Alois Piskala, Institute of Organic Chemistry, Czechoslovak Academy of Sciences, Prague. DZNep was kindly provided by Dr. Victor E. Marquez, Chemical Biology Laboratory, National Cancer Institute, Frederick, MD, USA. 5-AZA-CdR and DZNep were dissolved in sterile phosphate buffer saline (PBS) pH 6.8 solution. GSK-126 and GSK-343 were dissolved in DMSO and obtained from Xcess Biosciences Inc. or Structural Genomics Consortium (Toronto, ON, Canada), respectively.

### Antineoplastic Assay

The *in vitro* antineoplastic activity of the drugs was evaluated by reduction of colony formation after drug treatment. The HL-60 and AML-3 cells were treated with the indicated concentrations of drugs. At the end of drug treatment, a cell count was performed using the Beckmann Model Z Coulter Counter. For colony assays, the cells were placed in 0.3% soft agar medium containing 20% serum. The number of colonies (>500 cells) was counted after 16–18 days of incubation. The cloning efficiency was in the range of 60%.

### Apoptosis Analysis

Annexin V and propidium iodide (PI) staining were used to assess apoptosis and was determined using flow cytometry. The cells were treated as indicated. Twenty-four hours after the end of drug treatment, the cells were washed twice with cold PBS and resuspended in 1× Annexin V binding buffer (BD Biosciences Pharmingen). Then, 2 × 10^5 ^cells were mixed gently with Annexin V-FITC (BD Biosciences Pharmingen) and PI solution (Sigma-Aldrich) and incubated for 15 min in the dark at room temperature. The cells were suspended in 1× Annexin V binding buffer, and staining was immediately quantified using a BD LSR Fortessa flow cytometer (San Jose, CA, USA) and analyzed with the BD DIVA (San Jose, CA, USA) software program. A minimum of 10,000 cells within the gated region was analyzed per measurement.

### Analysis of Gene Expression

Drug treatment of the HL-60 leukemic cells was performed as described previously (33). At 24 h after the end of drug treatment, total RNA was isolated from HL-60 cells using the RNeasy Plus Mini kit (Qiagen). Quantity and integrity of total RNA were checked with a 2100 Bioanalyzer instrument (Agilent). All samples had an RNA integrity number >8. Paired-end RNASeq libraries were constructed using the TruSeq RNA Sample Prep kit v2 (Illumina). Quantification and quality control of RNASeq libraries were performed prior to sequencing using Illumina’s recommended protocols. Hundred base pairs of paired-end RNA sequencing were performed using eight samples per sequencing lane on the Illumina HiSeq 2000 platform at the Genome Quebec Innovation Centre, Montreal, Canada. Reads were assembled to a reference genome [hg19, European Hapmap (CEU) Major Allele release] using TopHat v1.3.2. The number of mismatches allowed per read was set to 2. PCR duplicates were removed using Picard-tools^1^ and non-properly paired and non-uniquely mapped reads were filtered out with SAMtools.^2^ Recalibration and local realignment was performed with GATK tools. BAM files were processed with Cufflinks to estimate isoform-level relative abundances and to perform differential expression analysis. Unsupervised analysis and hierarchical clustering was performed using JMP Genomics v6.0 (SAS Institute). The data are deposited in GEO (Gene Expression Omnibus) database under accession number GSE94344.

For comparative analysis of the expression of the transcripts, the genes were divided into different groups with similar biological functions (e.g., differentiation, senescence, apoptosis, etc.) using the gene lists of pathway-focused arrays from SA Biosciences.^3^ Gene function and comparison of expression of transcripts in normal human tissues was done using BioGPS (35) and Gene Card (36). The list of tumor suppressor genes (TSGs) and oncogenes was obtained from Zhao et al. (37) and UniProt Consortium (38).

### Ingenuity Pathway Analysis

To elucidate the probable downstream effects on biological functions of the observed gene expression changes, we used the downstream effects analysis approach implemented in Ingenuity Pathway Analysis. The algorithm uses the Ingenuity Knowledge Base and is based on a collection of experimental observations manually curated from the literature or other databases. Nodes and network edges from these data are used in combination with the observed gene expression changes to infer cause–effect relationships with a direction of the causal effect, i.e., either activating or inhibiting (39).

### Ethical Considerations

This study was exempted from ethical approval by the Research Ethics Board of CHU Sainte-Justine since no patient samples were used.

## Results

### Antineoplastic Action of 5-AZA-CdR Plus EZH2 Inhibitors on Myeloid Leukemic Cells

**Figure 1:**
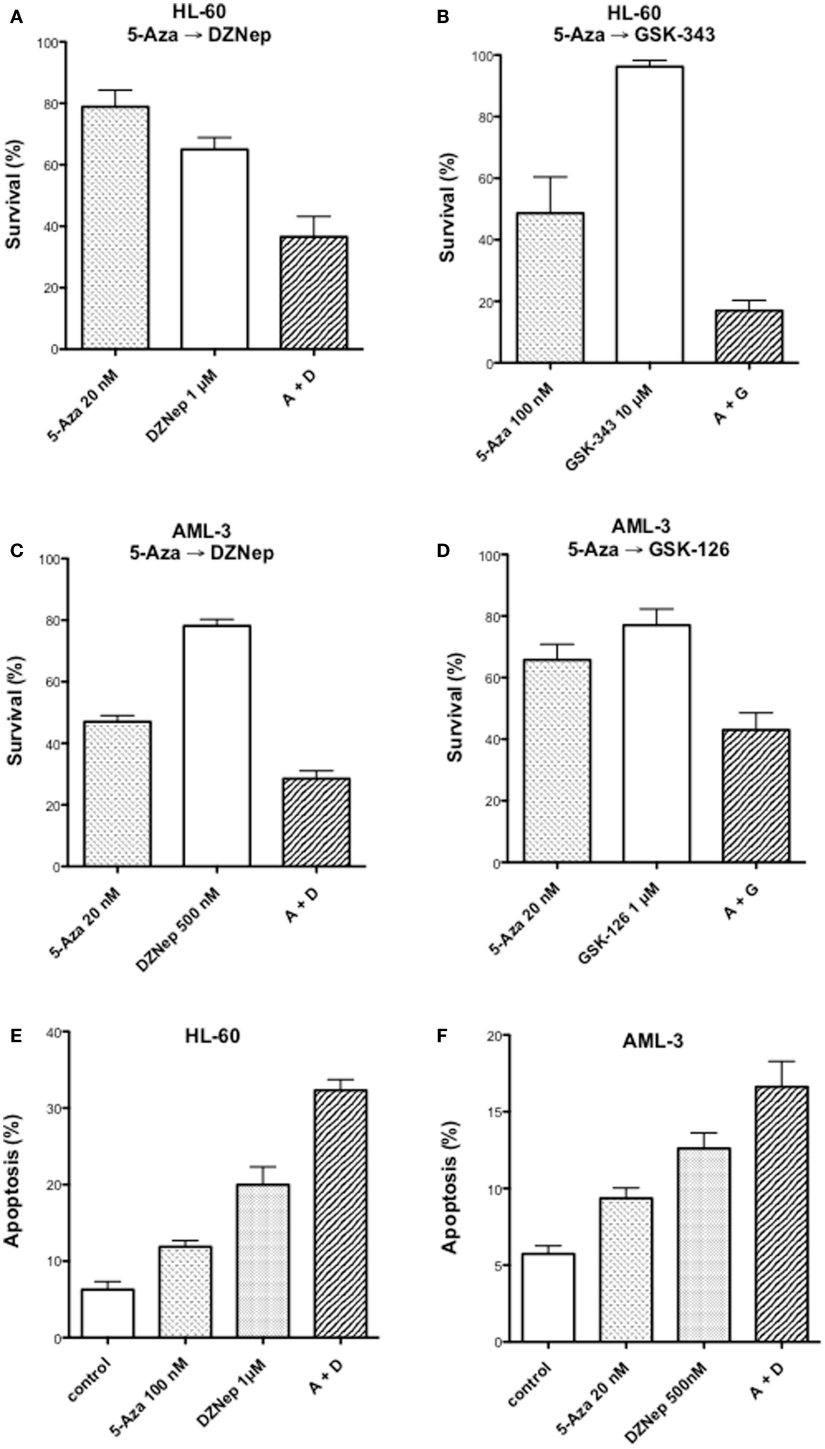
**Antineoplastic activity on myeloid leukemic cells after treatment with inhibitors of DNA methylation and EZH2**. Survival of human HL-60 **(A,B)** and AML-3 **(C,D)** leukemic cells after treatment with 5-Aza-CdR alone or in combination with inhibitors of EZH2: DZNep, GSK-343, or GSK-126. The cells were treated with the indicated concentration of 5-AZA-CdR, and, at 24 h, the different EZH2 inhibitors were added. At 48 h, the leukemic cells were placed in soft agar medium for colony assay to measure survival. Analysis of induction of apoptosis at 72 h after drug treatment in HL-60 **(E)** and AML-3 **(F)** leukemic cells. The results are expressed as mean ± SEM, *n* = 3. 5-AZA-CdR (5-Aza), DZNep (D), GSK-343 (G), GSK-126 (G).

We reported previously that the combination of 5-AZA-CdR and DZNep exhibited a synergistic antineoplastic effect on human HL-60 myeloid leukemic cells (33). In this report, we confirm that this synergy also takes place in human AML-3 myeloid leukemic cells as determined by a colony assay (Figures 1A,C). The AML-3 cells are more sensitive to 5-AZA-CdR than the HL-60 cells. For both cell lines, the combination of 5-AZA-CdR and DZNep showed a similar synergistic antileukemic effect. The antineoplastic action of DZNep on EZH2 is indirect and due to its potent inhibition of adenosyl homocysteine hydrolase (22). This action results in the accumulation of adenosyl homocysteine, a competitive inhibitor of S-adenosyl-l-methionine-dependent methylations. EZH2 uses S-adenosyl-l-methionine as the methyl donor to methylate H3K27. In support that the principle target for DZNep is EZH2, we compared its antileukemic action with the specific inhibitors of EZH2, GSK-126, and GSK-343 (40). We observed a similar synergy with all the combinations with these agents, suggesting that EZH2 is the primary target of DZNep (Figures 1B,D). However, DZNep was more active than GSK-343 or GSK-126 (Figures 1A,C). We also investigated the induction of apoptosis by 5-AZA-CdR and DZNep on HL-60 and AML-3 leukemic cells (Figures 1E,F). The combination showed a synergistic induction of apoptosis for both cell lines.

### Effect of 5-AZA-CdR and DZNep on Global Gene Expression in HL-60 Leukemic Cells

**Figure 2:**
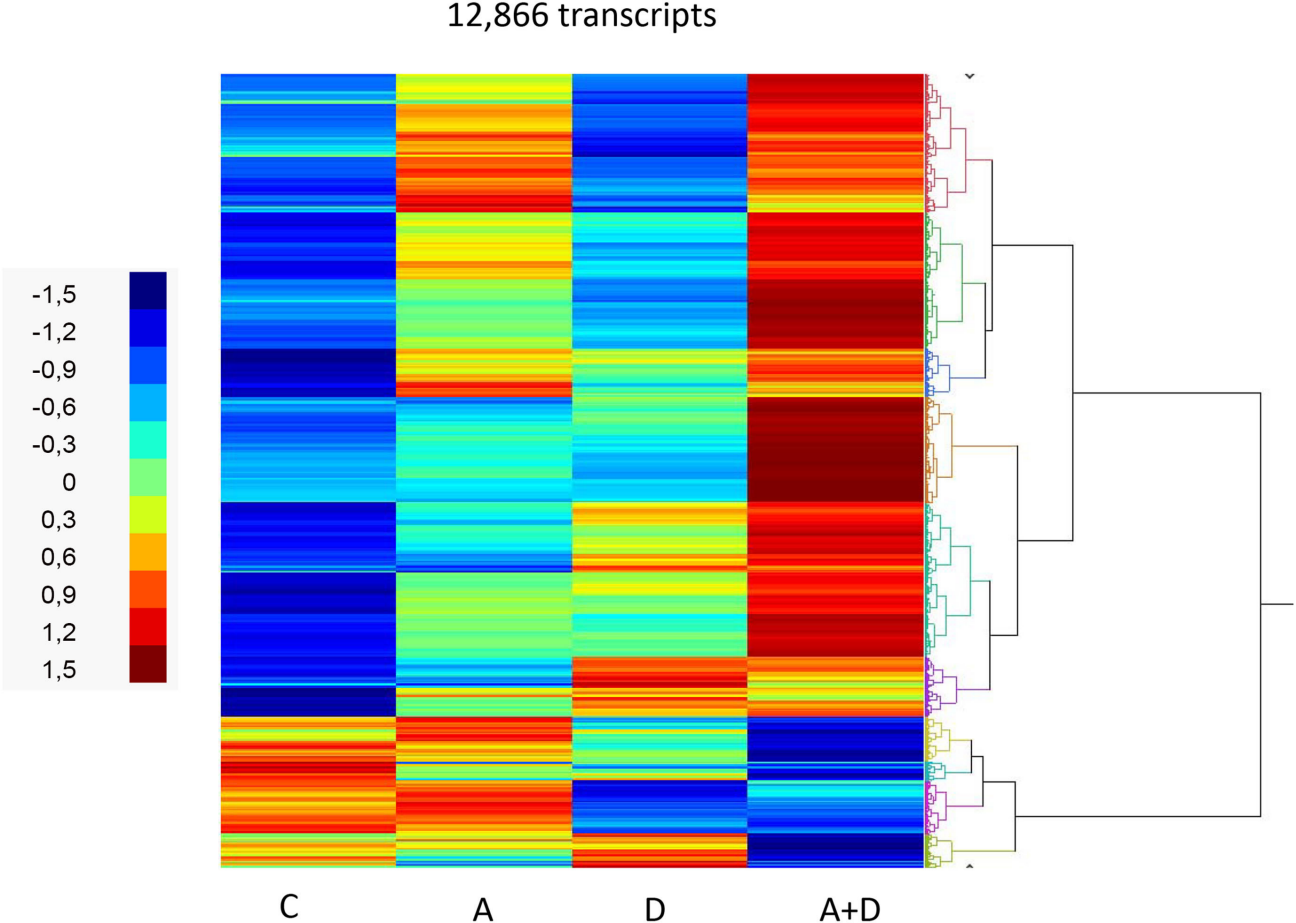
**RNA sequence analysis of global gene expression in HL-60 leukemic cells after treatment with 5-AZA-CdR and/or DZNep**. Heat map of expression of transcripts. HL-60 leukemic cells were treated with 100 nM 5-AZA-CdR (A). At 24 h 1,000 nM DZNep (D) was added to the medium. Drug removal at 48 h. RNA isolation at 72 h as described in Section “Materials and Methods.” The pairwise comparisons were control (C) versus A; C versus D; and C versus A + D.

**Figure 3:**
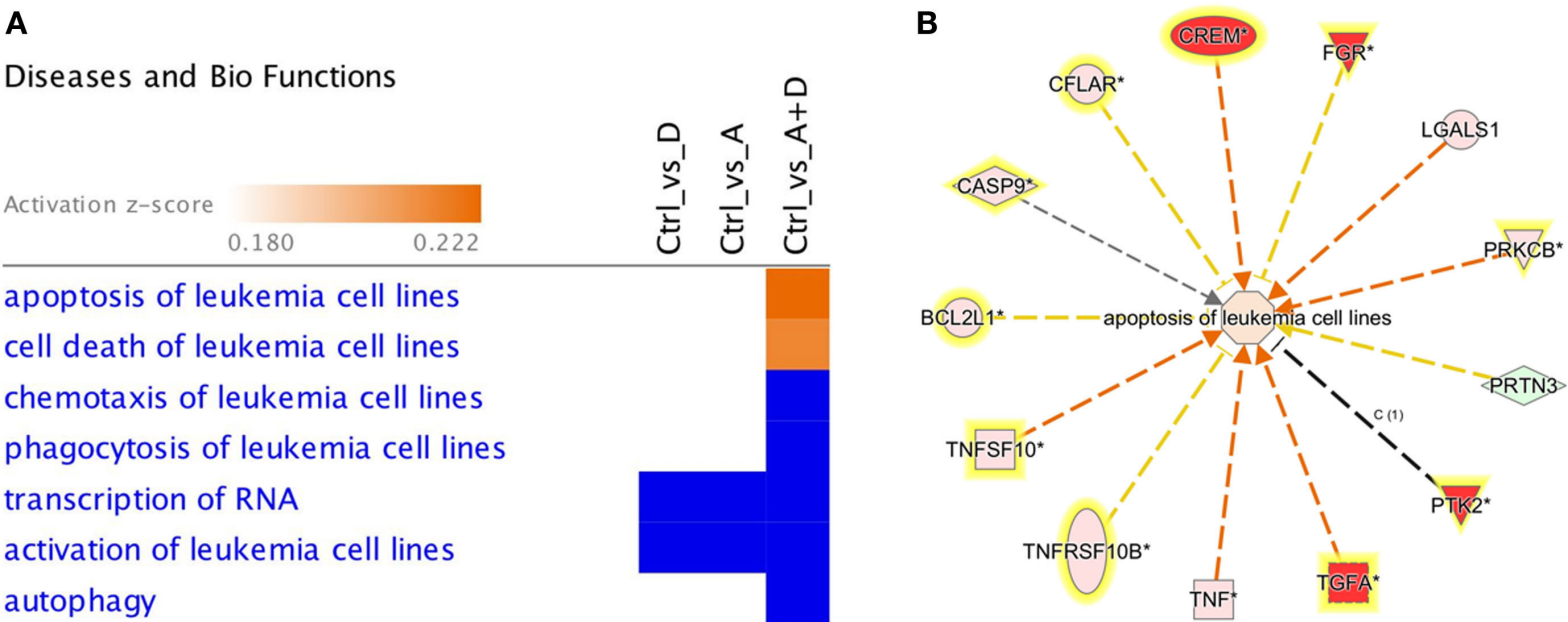
**Downstream effects analysis enables prediction of the effect of gene expression changes in each treatment**. **(A)** The heat map visualizes the downstream effects analysis results across the multiple treatments. The top clusters of diseases or biological functions that are predicted to increase or decrease in at least one treatment are shown. The disease or function scores are displayed using a gradient from dark blue to orange for activation *z*-scores. **(B)** The network of the top activated biological function in 5-AZA-CdR + DZNep treatment (apoptosis of leukemia cell lines) is shown. The edges are colored orange when leading to activation of the downstream node, blue when leading to its inhibition, and yellow if the findings underlying the relationship are inconsistent with the state of the downstream node. The predicted downstream effect of the observed expression changes on apoptosis is stronger pathway activation under the joint effect of 5-AZA-CdR and DZNep (activation *z*-score = 0.22).

The effect of 5-AZA-CdR and DZNep on global gene expression in HL-60 leukemic cells was determined by RNA sequence analysis (Figure 2). This figure shows the heat map displaying relative transcript abundances of 12,866 transcripts, each of which is differentially expressed (FDR 5%) at least in one of the pairwise comparisons of the four conditions. Relative to the control sample, 5-AZA-CdR alone showed a greater increase in the expression of transcripts as compared to DZNep. DZNep increased the activation of many cohorts of genes, but also showed a reduction in the expression of some genes. The combination of 5-AZA-CdR plus DZNep showed a synergistic increase in expression of hundreds of genes (Figure 2). Specific data on all of the genes are shown in Table S1 in Supplementary Material. In order to evaluate this very large data set, we used Ingenuity Pathway Analysis, an application for interpreting biological data and modeling complex networks (39). This analysis can identify the most relevant signaling pathways and has the potential to predict the downstream effects of the experimental chemotherapy. We performed a comparison analysis of the three pairwise contrasts (control versus 5-AZA-CdR, control versus DZNep, and control versus 5-AZA-CdR + DZNep) from the database generated from HL-60 leukemic cells treated with these epigenetic agents. The analysis revealed the clusters of biological functions that show increased or decreased activity across the different contrasts and enabled visualization across the three contrasts (Figure 3A). In particular, this analysis revealed the top most synergistically affected biological functions are apoptosis (activation *z*-score = 0.22 in 5-AZA-CdR + DZNep) and cell death (activation *z*-score = 0.18 in 5-AZA-CdR + DZNep) of leukemia cell lines. Based on expression levels of all genes implicated in these processes, both functions are predicted as highly activated under the synergistic treatment. The network of the top activated biological functions in 5-AZA-CdR + DZNep treatment (apoptosis of leukemia cell lines) is shown in Figure 3B. This conclusion was confirmed by our data on specific genes related to these phenomena as discussed in the section on apoptosis.

Below, we focused on the effect of treatment with 5-AZA-CdR and DZNep on gene expression levels of individual genes from several cohorts of genes based on their functional ontologies and in particular genes implicated in leukemogenesis, apoptosis and differentiation. Due to the relatively large number of genes whose expression was modulated by treatment with these epigenetic agents, we limited our focus to a subset of genes that are highly related to leukemogenesis (35–38). Nonetheless, Table S1 in Supplementary Material contains the extended list of specific genes affected by the action of 5-AZA-CdR plus DZNep.

### Effect of 5-AZA-CdR and DZNep on Expression of Differentiation Genes

**Figure 4:**
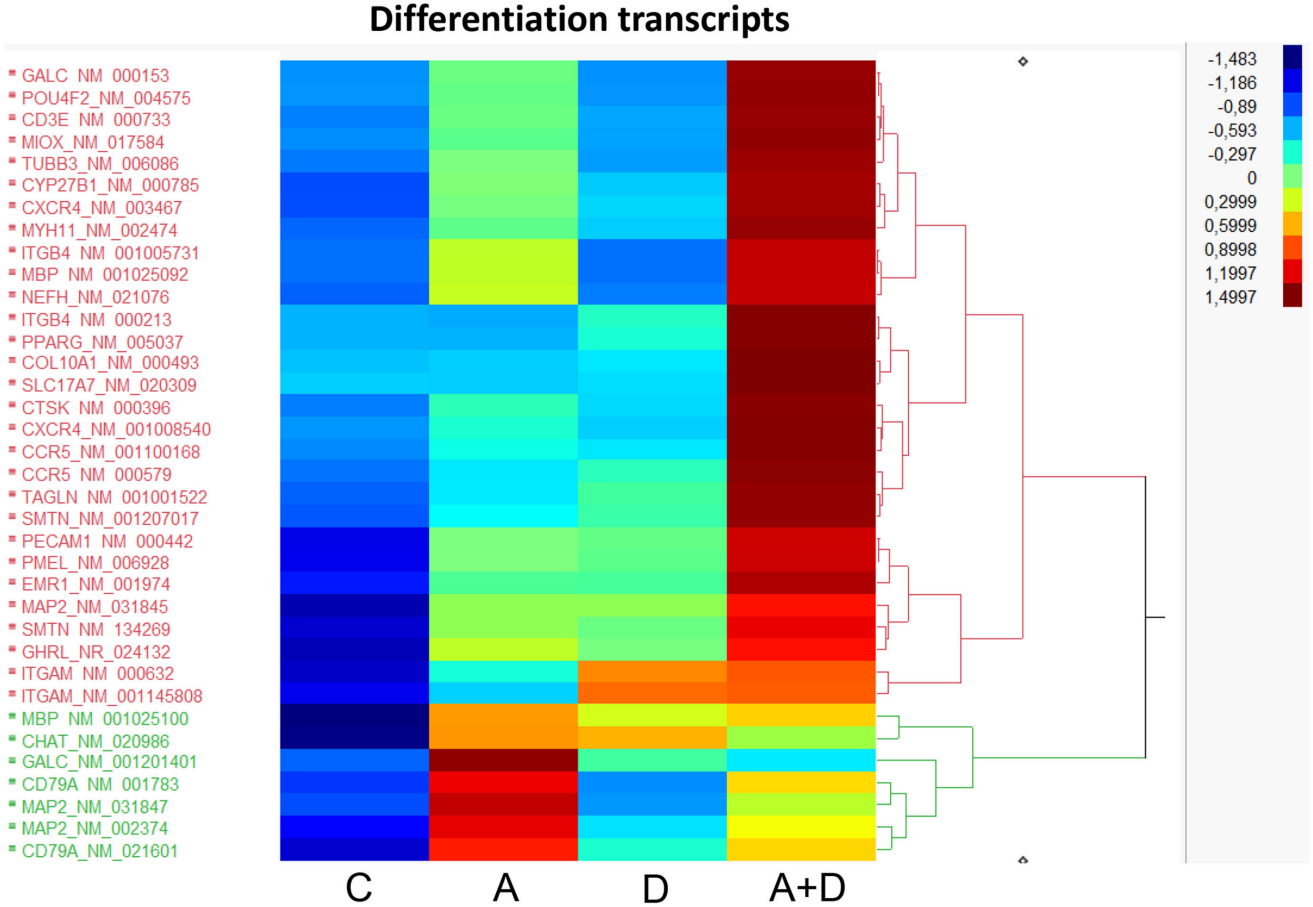
**Heat map expression of genes related to differentiation after 5-AZA-CdR and DZNep treatment of HL-60 myeloid leukemic cells**. Drug treatment of the leukemic cells is described in Figure 2. C, control; A, 5-AZA-CdR; D, DZNep; A + D, 5-AZA-CdR plus DZNep.

Since a block in differentiation is one of the hallmarks of AML, we investigated the action of this epigenetic therapy on the expression of genes that program differentiation. The transcriptional responses of genes related to differentiation after treatment with 5-AZA-CdR plus DZNep in HL-60 leukemic cells are shown in Table 1 and Figure 4. Many of the genes involved in differentiation were activated, some of which showed a remarkable synergistic activation by this combination of epigenetic agents. The expression of these genes is observed primarily in differentiated tissues.^4^ *NEFH* gene codes for neurofilaments that are normally expressed in neuronal cells. *MYH11* gene codes for myosin and exhibits high expression in smooth muscle. *PMEL*, a premelanosome gene, is expressed predominantly in the retina. These genes are important markers for cellular differentiation. These data indicate that 5-AZA-CdR plus DZNep turn on several differentiation genes that are not related to the phenotype of myeloid leukemic cells and may contribute to the antileukemic action of these epigenetic agents. 5-AZA-CdR plus DZNep also exhibited a synergistic activation of many genes involved in development (Table S2 in Supplementary Material).

**Table 1:** **Relative expression for differentiation-related genes in HL-60 leukemic cells after treatment with 5-AZA-CdR and DZNep**.

Transcript	Genbank no.	Fold change relative to control		
		5-AZA-CdR	DZNep	5-AZA-CdR + DZNep
CCR5	NM_001100168	16.3	11.7	71.7
NEFH	NM_021076	36.3	3.9^a^	59.0
EMR1	NM_001974	21.4	20.3	41.6
EGR1	NM_001964	5.2	0.3	27.5
CXCR4	NM_001008540	3.0	0.7^a^	23.0
MYH11	NM_002474	8.8^a^	3.4^a^	21.1
ITGAM	NM_001145808	6.2	17.9	19.4
PECAM1	NM_000442	5.3	4.3	19.2
PMEL	NM_006928	10.6^a^	9.1^a^	19.0
CTSK	NM_000396	3.2	1.1	18.9
COL10A1	NM_000493	0.1^a^	0.5^a^	17.0
SMTN	NM_134269	8.6	6.6	16.9
SLC17A7	NM_020309	0.005^a^	0.04^a^	15.4
ITGAM	NM_000632	5.2	12.5	14.7
PPARG	NM_005037	0.001^a^	1.5^a^	13.8
CXCR4	NM_003467	1.8	0.3	13.3
TUBB3	NM_006086	3.1	0.04^a^	13.1
MAP2	NM_031845	5.7	5.2	9.4
ITGB4	NM_000213	0.02^a^	0.7^a^	9.0
CYP27B1	NM_000785	1.2	0.2^a^	5.0
TAGLN	NM_001001522	0.2^a^	0.4^a^	3.5
CD79A	NM_021601	4.9	1.0	3.3

*^a^Not significant*.

### Effect of 5-AZA-CdR and DZNep on Expression of Genes Related to Apoptosis and Senescence

**Figure 5:**
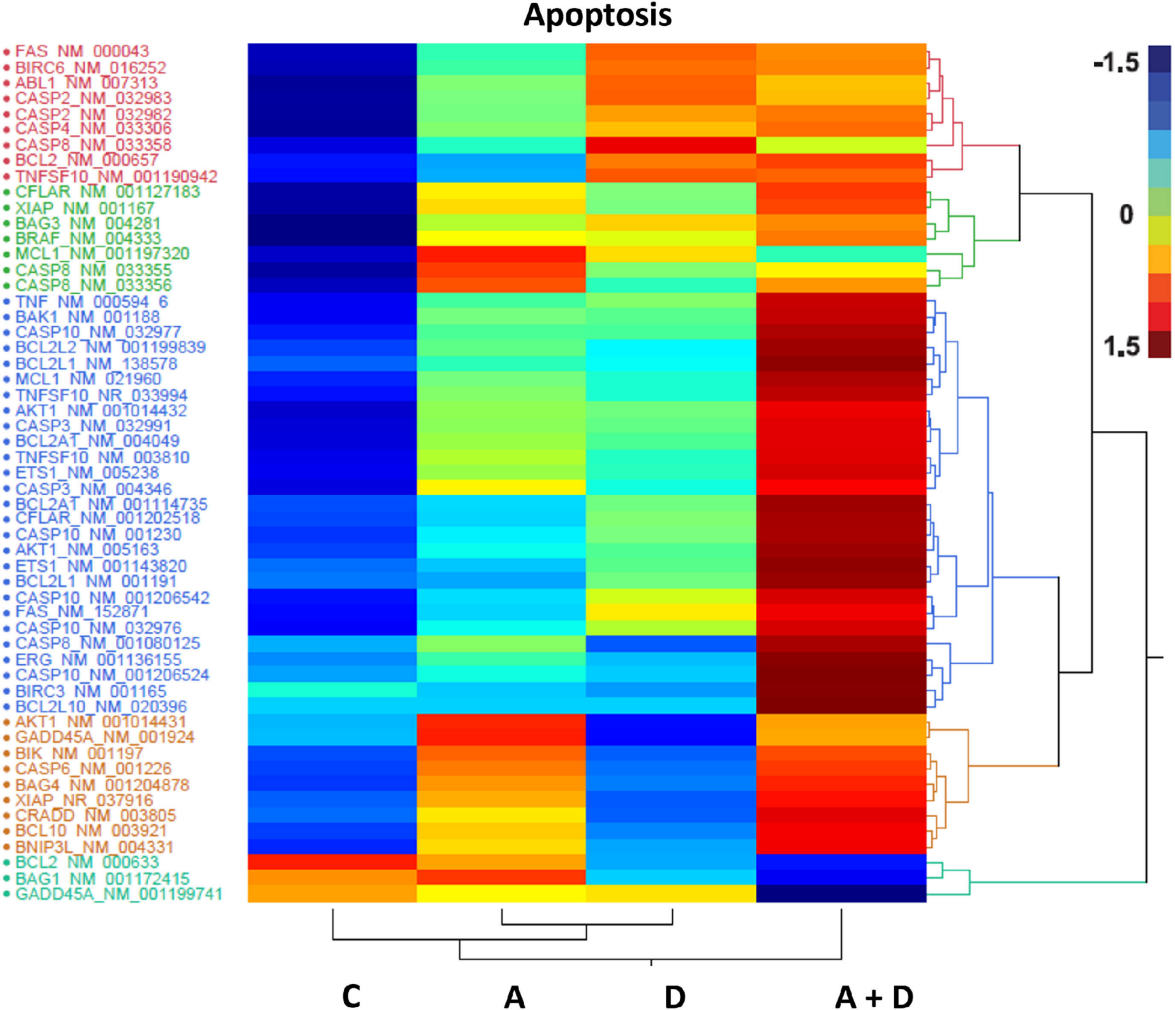
**Heat map expression of genes related to apoptosis after 5-AZA-CdR and DZNep treatment of HL-60 myeloid leukemic cells**. Drug treatment of the leukemic cells is described in Figure 2. C, control; A, 5-AZA-CdR; D, DZNep; A + D, 5-AZA-CdR plus DZNep.

The individual gene data on the synergistic activation of apoptosis-related genes in HL-60 leukemic cells by 5-AZA-CdR and/or DZNep are shown in Table 2 and Figure 5. The combination of 5-AZA-CdR and DZNep exhibited an increase of the expression of many genes that are positive regulators of apoptosis, such as *TNFSF10, CASP6, CASP10, BNIP3L*, and *GADD45A* (41). However, the combination also exhibited the activation of expression of several antiapoptotic genes, such as *BCL2A1, BCL2L1*, and *XIAP* (41). The action of 5-Aza-CdR and DZNep on the induction of apoptosis in HL-60 and AML-3 leukemic cells (Figures 1E,F) indicates activation of proapoptotic genes is the predominant action of these epigenetic agents and is clearly supported by our Ingenuity Pathway Analysis described above (Figure 3).

**Table 2:** **Relative expression for apoptosis-related genes in HL-60 leukemic cells after treatment with 5-AZA-CdR and DZNep**.

Transcript	Genbank no.	Fold change relative to control		
		5-AZA-CdR	DZNep	5-AZA-CdR + DZNep
TNF	NM_000594_6	13.6	17.2	45.2
BIK	NM_001197	41.6	0.6	43.8
ERG	NM_001136155	14.8	4.8	38.3
ETS1	NM_005238	13.7	6.8	37.0
BCL2A1	NM_004049	10.8	7.0	23.7
TNFSF10	NM_033994	12.1	8.0	22.9
BCL2A1	NM_001114735	3.3	6.8	19.7
FAS	NM_152871	4.4	10.3	15.6
BCL2L1	NR_001191	0.3	3.1	13.3
CASP10	NM_032976	3.6	6.3	12.2
GADD45A	NM_001199741	0.05	0.08	11.7
CFLAR	NM_001202518	1.3	3.1	9.0
TNFSF10	NM_003810	2.2	0.8	6.0
CASP10	NM_001206542	0.9	2.6	6.0
CASP10	NM_032977	0.9	0.7	4.8
BNIP3L	NM_004331	2.2	0.07	4.8
CASP10	NM_001206524	0.2	0.004	4.0
CASP10	NM_001230	0.2	0.5	3.3
CASP6	NM_001226	2.6	0.001	3.2
XIAP	NR_037916	2.0	0.03	3.0
XIAP	NM_001167	2.0	0.9	3.0

**Figure 6:**
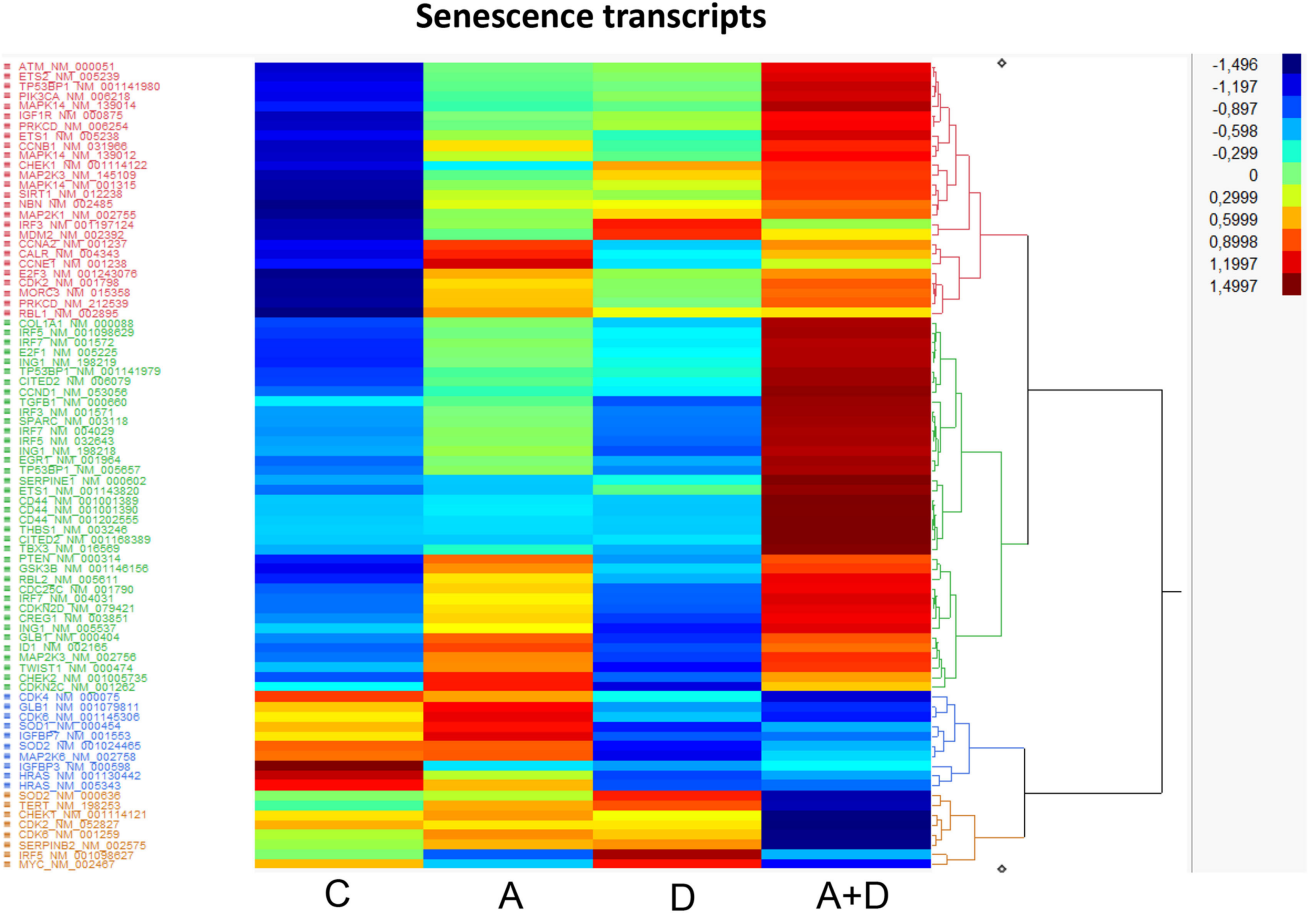
**Heat map expression of genes related to senescence after 5-AZA-CdR and DZNep treatment of HL-60 myeloid leukemic cells**. Drug treatment of the leukemic cells is described in Figure 2. C, control; A, 5-AZA-CdR; D, DZNep; A + D, 5-AZA-CdR plus DZNep.

Cellular senescence is the phenomenon by which normal cells cease to divide after threshold limit of cell divisions. This phenomenon is known as “replicative senescence,” or the Hayflick limit (42). Suppression of senescence can favor the development of malignancy (43). The treatment of HL-60 leukemic cells with 5-AZA-CdR and/or DZNep leads to the synergistic activation of many genes related to senescence (Table 3; Figure 6). Several of the activated genes are initiators of senescence, including *SPARC, EGR1, SERPINE1*, and *TP53BP1*. The activation of these genes by 5-AZA-CdR and/or DZNep may play an important role with respect to the antineoplastic action on HL-60 leukemic cells.

**Table 3:** **Relative expression for senescence related genes in HL-60 leukemic cells after treatment with 5-AZA-CdR and DZNep**.

Transcript	Genbank no.	Fold change relative to control		
		5-AZA-CdR	DZNep	5-AZA-CdR + DZNep
CD44	NM_001001391	317.4^a^	0.001^a^	488.9^a^
THBS1	NM_003246	1.9^a^	5.3^a^	65.9
TP53BP1	NM_005657	21.8	0.4^a^	47.0
ETS1	NM_005238	13.7	6.8	37.0
SERPINE1	NM_000602	2.4^a^	7.4^a^	34.2
EGR1	NM_001964	5.2	0.3	27.5
SPARC	NM_003118	6.9	3.4^a^	19.2
IRF5	NM_032643	6.5	0.001^a^	17.4
ID1	NM_002165	16.9	1.3^a^	16.3
CDC25C	NM_001790	8.6	0.003^a^	14.2
CHEK1	NM_001114122	2.4	8.5	12.0
IRF5	NM_001098629	3.2	1.4	10.9
CDKN2D	NM_079421	3.9	0.07^a^	9.5
IRF7	NM_004029	3.1^a^	0.3^a^	9.5
IGFBP3	NM_000598	12.0^a^	0.001^a^	9.0
IRF7	NM_001572	2.5	0.7	8.6
CDK6	NM_001145306	0.8	2.5	6.4
CDKN2D	NM_001800	1.5^a^	9.8^a^	6.0^a^
CCND1	NM_053056	0.3^a^	0.09^a^	5.6
IRF7	NM_004031	2.4	0.06^a^	4.8
CITED2	NM_006079	0.5	0.1	4.2
COL1A1	NM_000088	1.2	0.2^a^	3.8
ING1	NM_198218	0.9^a^	0.4^a^	3.7
MAPK14	NM_139014	0.8^a^	0.8^a^	3.6

*^a^Not significant*.

### Effect of 5-AZA-CdR and DZNep on Expression of TSGs

5-AZA-CdR and DZNep exhibited a synergistic activation of the expression of several important TSGs in HL-60 leukemic cells (Table 4). For example, *CDKN2B* (p15) is a cyclin-dependent kinase (CDK) inhibitor that inhibits cell growth by blocking G1 progression into S phase. *CDKN2B* function as a TSG in AML is well established (44). Hypermethylation of CpG islands in its promoter region was shown to occur frequently (45). This epigenetic state is associated with reduced expression (46) and is an important molecular predictor of outcome. Loss of expression of *CDKN2B* accelerates the development of myeloid leukemia in transgenic mice (47). Another important TSG is *CDKN1A* (p21), the expression of which is correlated with cell-cycle arrest that precedes terminal differentiation in a variety of tissues (48). *CDKN1A* is also implicated in regulation of cell growth and cell response to DNA damage. In response to DNA damage, p53 induces *CDKN1A* expression, which is responsible for the cell-cycle arrest at the G1 checkpoint (48). Other TSGs show similar patterns. We observed massive overexpression of *EGR1*, a zinc finger protein that functions as a transcriptional regulator. Overexpression of *EGR1*, one of the derepressed genes in EZH2-deficient leukemic cells, promoted profoundly the differentiation of AML cells (49). *EXT1* (Exostosin-1), a glycosyltransferase, is another highly upregulated gene whose expression is reduced in human cancer cells by DNA methylation (50). Introduction of a functional *EXT1* gene into cancer cell lines induces tumor-suppressor-like features, such as reduced colony formation and tumor growth in nude mouse xenografts. DNA methylation of *EXT1* is common in leukemia (50). *HES1*, a helix-loop transcriptional repressor, is frequently downregulated in AML (51). *HES1* activation suppresses proliferation of leukemia cells in AML. These results demonstrate the TSG role of *HES1* in AML. Finally, we observe upregulation of *RUNX3* a runt-domain transcriptional factor. The hypermethylation of this gene is frequent in AML cell lines (52). Relapse-free survival of AML patients without *RUNX3* methylation was significantly better than that of methylated cases. These results support the idea that *RUNX3* silencing is an important event in AML.

**Table 4:** **Expression of tumor suppressor genes in HL-60 leukemic cells after treatment with 5-AZA-CdR and DZNep**.

Transcript	Genbank no.	Fold change relative to control		
		5-AZA-CdR	DZNep	5-AZA-CdR + DZNep
AIM	NM_004833	23.3	18.3	63.3
BTG2	NM_006763	21.1	5.4	64.6
CDKN1A	NM_000389	17.7	9.8	43.4
CDKN2B	NM_004936	4.1	1.6	40.6
CD82	NM_002231	29.4	18.8	57.3
CDH5	NM_015557	14.2	14.9	70.2
DAB2IP	NM_138709	11.7	3.0	64.3
DAPK2	NM_014326	16.0	2.9	36.4
EGR1	NM_001964	5.2	0.26	27.5
EMP1	NM_001423	4.7	17.9	28.1
ERRFI1	NM_018948	31.5	3.6	75.5
ETS1	NM_005238	13.7	6.8	37.0
EXT1	NM_000127	9.0	1.0	41.2
HES1	NM_005524	19.7	0.6	71.8
JUP	NM_002230	43.5	3.5	80.9
NR4A3	NM_006981	3.3	3.0	42.6
PLCB1	NM_015192	37.9	5.5	59.2
PTPRK	NM_002844	45.5	3.9	65.6
RUNX3	NM_001031680	5.9	0.5	30.6
SFN	NM_006142	22.8	5.2	46.8
THBS1	NM_003246	2.0	5.3	65.9
TP53INP1	NM_033285	23.8	9.0	46.3

## Discussion

Epigenetic alterations that silence gene expression play an important role in the development of AML. Aberrant DNA methylation that silences genes which suppress leukemogenesis occurs frequently in AML (45). The importance of this epigenetic modification is illustrated by the clinical activity of the potent inhibitor of DNA methylation, 5-AZA-CdR, for the treatment of AML (9). However, 5-AZA-CdR therapy of AML for most patients is not curative, and new approaches are needed to optimize the treatment of AML with this epigenetic drug. One factor that may limit the clinical effectiveness of 5-AZA-CdR for the treatment of AML is that this cytosine nucleoside analog has difficulty to activate DNA-methylated genes that contain the histone repressive marker, H3K27me3 (26). This latter epigenetic alteration is catalyzed by EZH2 methyltransferase (53). DZNep, a potent inhibitor of EZH2, shows remarkable antineoplastic activity against AML cells (25). This observation provided a good rationale to use DZNep in combination with 5-AZA-CdR. We reported previously that inhibition of DNA methylation and EZH2 by 5-AZA-CdR and DZNep, respectively, exhibits a synergistic antineoplastic effect on HL-60 AML cells (33). We confirm in this study that the synergistic antineoplastic action of this combination is also observed with a second human myeloid leukemic cell line, AML-3. DZNep is a global inhibitor of histone methylation (23). GSK-126 and GSK-343, specific inhibitors of EZH2, exhibit a similar synergy in combination with 5-AZA-CdR as observed with DZNep. This observation supports that EZH2 is the preferential target for DZNep.

In a previous study using real-time PCR and microarray analysis, we observed that 5-AZA-CdR plus DZNep exhibited a synergistic activation of several TSGs in HL-60 leukemic cells (33). In this report, we investigated the action of this combination on gene expression in greater depth using RNA sequence analysis. Each methodology has particular methodologically related detection biases and limitations, which could explain why some differences were observed between our previous microarray and RT-PCR results and the present RNA sequencing with respect to the expression of several of genes that were detected. Ideally for genes of interest, our RNASeq data would have been confirmed using a second methodology, but there was insufficient mRNA available to do this. However, we nevertheless observed a synergistic increase in expression of hundreds of different transcripts by this combination of epigenetic agents. This synergistic effect of 5-AZA-CdR plus DZNep is highly consistent with the antineoplastic action of this combination. Epigenetic activation of specific genes that program apoptosis are likely one of the major contributors to the antileukemic action of 5-AZA-CdR and DZNep (Figure 5). This is shown by a synergistic activation of several genes that induce apoptosis, such as *CASP6* (Table 2). These gene expression data are also supported by the synergistic induction of apoptosis in the HL-60 leukemic cells by 5-AZA-CdR plus DZNep (Figures 1E,F).

The genes that program differentiation also merit serious consideration (Table 1; Figure 4). These genes show a high frequency of DNA methylation in many different types of cancer and contain high levels of the H3K27me3 marker in adult stem/progenitor cells (27). The synergistic activation of so many genes that program differentiation by 5-AZA-CdR plus DZNep support the hypothesis that gene silencing by DNA methylation and H3K27me3 plays a very important role in the leukemogenesis of AML. Since these epigenetic alterations in the leukemic cells resemble that of embryonic stem cells (54, 55), it is possible that this epigenetic therapy has the potential to target leukemic stem cells (32). This interesting hypothesis merits further investigation. This may be a one of the key mechanisms that explains the block in differentiation that is a hallmark of AML. The synergistic activation of genes that promote senescence may also contribute to its antileukemic action of this combination of these epigenetic agents.

It is of interest to investigate if the antineoplastic synergy that we observed between 5-AZA-CdR and DZNep on myeloid leukemic cells also occurs in tumors. In this regard, we reported recently that 5-AZA-CdR and DZNep show a synergistic antineoplastic action against human lung carcinoma cells (56). The synergistic interaction between these two epigenetic agents was also observed against rhabdoid tumor cells (57). 5-AZA-CdR in combination with the EZH2 inhibitor, GSK-126, inhibited the *in vivo* growth of a prostate tumor xenograft more effectively than treatment with 5-AZA-CdR alone (58). These observations suggest that the reversal of gene silencing by DNA and histone methylation using epigenetic agents may also be an effective therapy for tumors.

## Conclusion

Our data support the hypothesis that the antileukemic activity of 5-AZA-CdR plus DZNep results from the summation of the activation of different cohorts of genes that suppress leukemogenesis by different mechanisms, including the induction of apoptosis, differentiation, development, and senescence. However, since so many genes in these different categories are activated, it is not possible to define the precise mechanisms involved. The genes that program differentiation may play a prominent role in this process since the block in differentiation is one of the hallmarks of AML. In order to accomplish this, leukemic stem cells have to “turn off” the genes that program differentiation by DNA and H3K27 methylation. The synergistic activation of so many genes that suppress malignancy by 5-AZA-CdR plus DZNep suggests that epigenetic gene silencing by DNA and histone methylation plays a major role in leukemogenesis. 5-AZA-CdR plus DZNep have the potential to reverse this “double lock” gene silencing mechanism and thus target leukemic stem cells (32). This interesting epigenetic therapy merits clinical investigation in patients with advanced myeloid leukemia.

## Supporting Information

The dataset supporting the conclusions of this article is available in the BioProject database of the National Center for Biotechnology Information (NCBI) (BioProject ID: PRJNA316529; http://www.ncbi.nlm.nih.gov/bioproject/316529).

## Author Contributions

RM, YI, and SC conceived and designed the experiments; RM, SC, and LM performed the experimental work. RM, SC, and YI analyzed the data. All the authors read and approved the manuscript.

## Conflict of Interest Statement

The authors declare that the research was conducted in the absence of any commercial or financial relationships that could be construed as a potential conflict of interest. The reviewer AC and handling Editor declared their shared affiliation, and the handling Editor states that the process nevertheless met the standards of a fair and objective review.

## Footnotes

^1^http://picard.sourceforge.net/.

^2^http://samtools.sourceforge.net/.

^3^www.sabiosciences.com.

^4^http://biogps.org.

## Supplementary Material

Table S1 **RNA sequence analysis of global gene expression in HL-60 leukemic cells after 5-AZA-CdR (A) and/or DZNep (D)**.

Table S2 **Developmental genes that show frequent DNA hypermethylation in cancer: relative expression after treatment of HL-60 leukemic cells with 5-AZA-CdR and DZNep**.
