# Supplementary material for: Inhibition of DNA and Histone Methylation by 5-Aza-2′-Deoxycytidine (Decitabine) and 3-Deazaneplanocin-A on Antineoplastic Action and Gene Expression in Myeloid Leukemic Cells
Source: Front Oncol. 2017 Feb 15;7:19. doi: 10.3389/fonc.2017.00019 (PMC5309231; doi:10.3389/fonc.2017.00019)
Supplement: Table S2 — Developmental genes that show frequent DNA hypermethylation in cancer: relative expression after treatment of HL-60 leukemic cells with 5-AZA-CdR and DZNep. [file Table_2.PDF]

TABLE S2 | Developmental genes that show frequent DNA hypermethylation in cancer*: Relative expression after treatment of HL60 leukemic cells with 5-AZA-CdR and DZNep

		Fold change relative to control		
**Transcript**	**Genbank no.**	**5-Aza-CdR**	**DZNep**	**5-AzaCdR + DZNep**
**ABCA1**	NM_005502	2.5	0.56	5.3
**ACSL1**	NM_001995	1.4	0.55	4.1
**ACVRL1**	NM_001077401	14.3	6.15	56.0
**ADAM12**	NM_003474	0.5	4.0	24.5
**ADRB2**	NM_000024	5.5	2.9	11.6
**AGPAT9**	NM_0032717	2.6	2.1	12.8
**APBB1**	NM_145689	4.6	11.1	31.8
**ARHGAP21**	NM_020824	10.9	0.01	21.2
**ARHGEF17**	NM_014786	4.3	0.08	6.7
**ARL4C**	NM_005737	6.9	1.46	22.7
**BIK**	NM_001197	41.6	0.62	43.8
**BTG2**	NM_006763	21.1	5.43	64.6
**CAMK2B**	NM_172081	30.6	0.04	77.8
**CAMK2D**	NM_172115	7.3	5.7	7.69
**CAMK4**	NM_001744	0.06	0.03	6.33
**CCNA1**	NM_003914	21.8	30.4	65.0
**CD70**	NM_001252	3.15	4.52	7.60
**CD9**	NM_001769	5.44	0.19	12.3
**CDKN2B (p15)**	NM_004936	4.11	1.56	40.7
**CDO1**	NM_001801	17.8	<1	33.1
**CITED1**	NM_004143	0.42	285	8.84
**CKB**	NM_001823	14.4	0.34	31.0
**COL4A1**	NM_001845	3.33	0.95	5.38
**COL9A2**	NM_001852	3.64	0.008	17.0
**DES**	NM_001927	18.0	0.39	51.8
**DGAT1**	NM_012079	3.69	2.79	15.1
**DKK2**	NM_014421	1.45	0.25	11.4
**DLL1**	NM_005618	9.03	1.03	49.6
**DNASE2**	NM_001375	12.8	1.84	28.5
**DNMT3A**	NM_022552	0.95	0.34	4.08
**DUSP10**	NM_144728	4.76	1.80	18.4
**DUSP6**	NM_001946	1.53	0.44	8.40
**ECE1**	NM_001113348	0.78	0.19	3.71
**EGR1**	NM_001964	5.18	0.26	27.5
**EPHA4**	NM_004438	3.6	2.64	44.8
**EPHB6**	NM_004445	39.3	3.64	85.0
**EPS8**	NM_004447	11.5	0.30	14.5
**ETS1**	NM_005238	13.7	6.79	37.0
**FST**	NM_006350	9.54	12.1	28.5

**GABRB2**	NM_000813	3.55	1.06	5.74
**GAD1**	NM_000817	9.50	1.12	18.0
**GALR2**	NM_003857	0.60	0.22	3.68
**GFI1**	NM_004188	0	0.91	15.2
**GNG13**	NM_016541	0.06	5.01	9.64
**GPR155**	NM_001033045	1.11	0.008	5.03
**GPR63**	NM_001143957	0.41	0.39	3.05
**GPRC5C**	NM_018653	8.1	2.62	34.7
**GRASP**	NM_00181711	30.7	1.12	50.0
**HES6**	NM_018645	12.1	1.42	14.4
**HIC1**	NM_006497	17.2	<1	36.6
**HTR7**	NM_019859	0.05	2.0	3.45
**ICAM1**	NM_000201	8.05	8.06	24.7
**IGFBP3**	NM_000598	12.0	<1	9.05
**IL17RA**	NM_014339	0.97	0.40	3.73
**INHBB**	NM_002193	3.14	0.48	24.4
**IRF8**	NM_002163	1.03	0.49	10.8
**ITGA2**	NM_002203	10.2	0.59	32.9
**ITPKB**	NM_002221	0.55	0.13	5.95
**KAL1**	NM_000216	17.4	0.16	24.1
**KCNH2**	NM_000238	56.1	4.71	124.3
**KIFC3**	NM_001130100	7.50	5.57	30.2
**KITLG**	NM_003934	15.8	210	23.0
**KLF4**	NM_004235	0.62	0.63	3.67
**KLF5**	NM_001730	1.83	0.95	5.14
**LRP12**	NM_0013437	0.36	0.94	3.05
**LTBP3**	NM_021070	1.64	0.12	68.2
**MAPK11**	NM_002751	0.51	0.20	3.82
**MAPK13**	NM_002754	8.08	1.32	13.0
**MATK**	NM_139354	3.90	14.0	33.7
**MCAM**	NM_006500	9.71	2.06	25.8
**MYH11**	NM_002474	8.79	3.35	21.1
**NEFH**	NM_021076	36.3	3.93	58.9
**NFATC1**	NM_172387	0.10	1.35	2.95
**NGFR**	NM_002507	2.71	0.22	20.8
**NPB**	NM_145039	0.31	5.87	3.20
**NR2F2**	NM_001145156	0.14	0.72	2.86
**NRP1**	NM_003873	3.77	4.06	23.8
**NXPH4**	NM_007224	2.41	1.18	19.3
**OTP**	NM_032109	103	3.64	97.4
**PAG1**	NM_018440	0.48	1.29	7.12
**PAQR8**	NM_133367	2.12	1.69	3.15
**PCGF2**	NM_007144	13.4	0.06	33.0
**PDE3A**	NM_000921	8.90	0.39	43.2

**PDGFB**	NM_033016	0.03	0.80	27.2
**PER3**	NM_016831	9.88	0.004	13.3
**PHLDA2**	NM_003311	4.87	0.32	8.97
**PKDREJ**	NM_006071	1.84	6.85	21.9
**PLCB1**	NM_015192	37.9	5.48	58.2
**PPARA**	NM_001001928	12.3	4.99	25.2
**PPARG**	NM_005037	0.001	1.50	13.8
**PPP1R16B**	NM_015568	1.69	3.79	15.8
**PRKCH**	NM_006255	2.27	0.01	38.5
**PTPRE**	NM_006504	0.71	0.65	10.6
**RASD1**	NM_016084	14.5	0.09	50.3
**RASSF1**	NM_170712	1.17	0.01	3.20
**RASSF5**	NM_182664	0.15	0.14	3.15
**RET**	NM_020975	24.5	0.004	48.2
**ROR2**	NM_004560	8.22	2.19	11.1
**S100A6**	NM_014624	30.4	0.84	45.1
**SARM1**	NM_015077	0.62	0.02	4.02
**SEMA7A**	NM_001146029	3.02	0.96	9.27
**SGCB**	NM_000232	2.26	0.26	3.31
**SLC17A7**	NM_020309	0.005	0.04	15.4
**SLC18A2**	NM_003054	0.99	0.27	5.27
**SLC1A4**	NM_001193493	0.85	2.20	3.42
**SMAD7**	NM_005904	1.20	0.46	8.33
**SMPD3**	NM_018667	45.2	0.31	82.2
**SNX18**	NM_052870	2.37	1.88	3.28
**SPHK1**	NM_182965	2.39	6.64	3.93
**SPOCK1**	NM_004598	1.19	0.05	4.97
**SPRY4**	NM_030964	0.02	0.44	4.98
**SSTR2**	NM_001050	2.92	3.59	31.9
**STBD1**	NM_003943	2.05	0.13	6.02
**SVIL**	NM_021738	10.2	1.78	21.1
**TNFRSF10D**	NM_003840	0.97	3.44	10.5
**TP73**	NM_005427	5.94	2.56	19.3
**TSSK3**	NM_052841	0.79	0.78	8.56
**VENTX**	NM_014468	20.3	0.02	76.9
**VGF**	NM_003378	0.64	0.36	4.41
**WNT10A**	NM_025216	0.03	0.41	3.30
**ZBTB16**	NM_001018011	2.56	0.12	19.2

*The gene list was obtained from Supplemental Table 7 of reference: Easwaran H et al. A DNA hypermethylation module for the stem/progenitor cell signature of cancer. Genome Res 2012;22:837-849.
